# Rapid Classification and Differentiation of Sepsis-Related Pathogens Using FT-IR Spectroscopy

**DOI:** 10.3390/microorganisms12071415

**Published:** 2024-07-12

**Authors:** Shwan Ahmed, Jawaher Albahri, Sahand Shams, Silvana Sosa-Portugal, Cassio Lima, Yun Xu, Rachel McGalliard, Trevor Jones, Christopher M. Parry, Dorina Timofte, Enitan D. Carrol, Howbeer Muhamadali, Royston Goodacre

**Affiliations:** 1 Centre for Metabolomics Research, Department of Biochemistry, Cell and Systems Biology, Institute of Systems, Molecular and Integrative Biology, University of Liverpool, Liverpool L69 7ZB, UK; shwan.ahmed@liverpool.ac.uk (S.A.); j.albahri@liverpool.ac.uk (J.A.); sahand.shams@liverpool.ac.uk (S.S.); cassio.lima@liverpool.ac.uk (C.L.); yun.xu@liverpool.ac.uk (Y.X.); 2 Department of Environment and Quality Control, Kurdistan Institution for Strategic Studies and Scientific Research, Sulaymaniyah, Kurdistan Region, Iraq; 3 Department of Pharmaceutical Chemistry, College of Pharmacy, King Khalid University, Abha 62529, Saudi Arabia; 4 Department of Veterinary Anatomy, Physiology and Pathology, Institute of Infection, Veterinary and Ecological Sciences, University of Liverpool, Neston CH64 7TE, UK; s.sosa-portugal@liverpool.ac.uk (S.S.-P.); tdorina@liverpool.ac.uk (D.T.); 5 Department of Clinical Infection, Microbiology and Immunology, Institute of Infection, Veterinary and Ecological Sciences, University of Liverpool, Liverpool L69 7BE, UK; rmcg@liverpool.ac.uk (R.M.); trjones@liverpool.ac.uk (T.J.); edcarrol@liverpool.ac.uk (E.D.C.); 6 Department of Clinical Sciences, Liverpool School of Tropical Medicine, Liverpool L7 8XZ, UK; christopher.parry@lstmed.ac.uk

**Keywords:** sepsis, FT-IR spectroscopy, bacterial classification, fingerprinting

## Abstract

Sepsis is a life-threatening condition arising from a dysregulated host immune response to infection, leading to a substantial global health burden. The accurate identification of bacterial pathogens in sepsis is essential for guiding effective antimicrobial therapy and optimising patient outcomes. Traditional culture-based bacterial typing methods present inherent limitations, necessitating the exploration of alternative diagnostic approaches. This study reports the successful application of Fourier-transform infrared (FT-IR) spectroscopy in combination with chemometrics as a potent tool for the classification and discrimination of microbial species and strains, primarily sourced from individuals with invasive infections. These samples were obtained from various children with suspected sepsis infections with bacteria and fungi originating at different sites. We conducted a comprehensive analysis utilising 212 isolates from 14 distinct genera, comprising 202 bacterial and 10 fungal isolates. With the spectral analysis taking several weeks, we present the incorporation of quality control samples to mitigate potential variations that may arise between different sample plates, especially when dealing with a large sample size. The results demonstrated a remarkable consistency in clustering patterns among 14 genera when subjected to principal component analysis (PCA). Particularly, *Candida*, a fungal genus, was distinctly recovered away from bacterial samples. Principal component discriminant function analysis (PC-DFA) allowed for distinct discrimination between different bacterial groups, particularly Gram-negative and Gram-positive bacteria. Clear differentiation was also observed between coagulase-negative staphylococci (CNS) and *Staphylococcus aureus* isolates, while methicillin-resistant *S. aureus* (MRSA) was also separated from methicillin-susceptible *S. aureus* (MSSA) isolates. Furthermore, highly accurate discrimination was achieved between *Enterococcus* and vancomycin-resistant enterococci isolates with 98.4% accuracy using partial least squares-discriminant analysis. The study also demonstrates the specificity of FT-IR, as it effectively discriminates between individual isolates of *Streptococcus* and *Candida* at their respective species levels. The findings of this study establish a strong groundwork for the broader implementation of FT-IR and chemometrics in clinical and microbiological applications. The potential of these techniques for enhanced microbial classification holds significant promise in the diagnosis and management of invasive bacterial infections, thereby contributing to improved patient outcomes.

## 1. Introduction

Sepsis is a life-threatening condition caused by an overwhelming response of the body to an infection. According to the Global Burden of Diseases (GBD) study in 2017, the worldwide occurrence of sepsis amounted to approximately 48.9 million cases annually, while the mortality rate attributed to sepsis was a staggering 11 million deaths, constituting 19.7% of the total global mortality [1]. Notably, these statistics indicate that the global estimates for sepsis provided by Rudd et al. are more than double the figures reported in previous research conducted by Fleischmann and colleagues [2]. Sepsis can arise from various infections, including bacterial, viral, and fungal. Respiratory tract infections, urinary tract infections, intra-abdominal infections, as well as infections affecting the skin and soft tissues are the prevalent sources of infection that have been associated with the development of sepsis [3]. A comprehensive knowledge and recognition of the diverse origins contributing to sepsis plays a vital role in formulating efficient strategies to prevent and manage this critical condition. In clinical diagnostics, identifying bacterial pathogens obtained from the human bloodstream, particularly in the context of sepsis, is crucial for guiding appropriate antimicrobial therapy and improving patient outcomes. Bacterial typing is a crucial process in microbiology that involves the identification and classification of bacterial strains based on their genetic, biochemical, or phenotypic characteristics. Traditional methods for bacterial identification have long been employed and relied heavily on culture-based techniques, but they often suffer from limitations such as time-consuming procedures, low sensitivity (blood cultures have a low sensitivity for the detection of bloodstream infections), and the need for specialised expertise. To overcome the limitations of culture-based methods, various diagnostic methods have been developed and employed to achieve accurate bacterial typing [4], such as enzyme-linked immunosorbent assay (ELISA) and polymerase chain reaction (PCR), which are known for their precision, high accuracy, and sensitivity. Nevertheless, despite their effectiveness, these methods also suffer from time-consuming procedures as well as the requirement for specialised personnel and substantial financial resources [5]. Nucleic acid sequencing, including 16S rRNA gene sequencing, provides an in-depth characterisation of bacterial genomes, enabling precise identification and classification. While these methods have shown promise in reducing turnaround times and enhancing sensitivity, they often require costly equipment, specialised bioinformatics training, and extensive infrastructure, limiting their widespread use, especially in resource-limited settings [6,7]. When it comes to identifying microorganisms at the species level, genotypic characterisation via 16S rRNA sequencing remains the most widely accepted and reliable method [8]. Whole-genome sequencing (WGS) shows significant promise in clinical diagnosis by offering precise species identification and the ability to achieve strain-level resolution. Its potential for routine testing in large hospitals has been confirmed as the cost of WGS continues to decline and serves as the gold standard [9].

Additionally, matrix-assisted laser desorption/ionisation time-of-flight mass spectrometry (MALDI-TOF-MS) has gained popularity as a rapid and cost-effective method for bacterial typing allowing for the rapid profiling of bacterial proteins and peptides, aiding in species-level identification. MALDI-TOF-MS identifies unique protein profiles or fingerprints generated by bacterial samples, enabling species identification and differentiation [10,11]. Mortier et al. carried out a comprehensive and broad benchmarking study to assess the effectiveness of MALDI-TOF-MS and machine learning methods for large-scale bacterial identification [12]. They employed datasets containing almost 100,000 spectra and more than 1000 different species. The extensive size and diversity of the data enabled a comparison across three noteworthy identification scenarios that are commonly undifferentiated in the literature: identifying novel biological replicates, novel strains, and novel species not present in the training data. The findings indicate that satisfactory identification rates were achieved in all three scenarios. The bacterial species-level identification offered by commercial MALDI-TOF-MS-based systems is hindered by their inability to effectively distinguish antimicrobial-resistant strains. Therefore, conventional methods must be employed to detect antimicrobial resistance after MALDI-TOF-MS analysis, resulting in a considerable time delay that significantly impedes the timely adjustment of antimicrobial therapies [13].

Vibrational spectroscopy approaches, including Raman and infrared, provide rich information about the biochemical composition of the cell, enabling the rapid and accurate identification of microorganisms. These techniques have seen numerous potential applications in exploring biological inquiries in recent years [5]. The initial published data demonstrated the capability of Raman microscopy, when combined with suitable chemometrics, to differentiate clinically relevant intact bacterial species [14]. The recent advancements in Fourier Transform Infrared (FT-IR) spectroscopy have shown great promise in revolutionising bacterial identification and discrimination [15]. IR and Raman spectroscopies, as standalones and combined into a single technology, have also been employed for the differentiation of bacterial species and for the detection of antimicrobial resistance (AMR) at the single-cell level [16,17]. Such methodologies enable the circumvention of the cultivation steps necessary in other techniques, thereby decreasing the time required for diagnosis. Suntsova et al. demonstrated the application of FT-IR spectroscopy as a viable approach for identifying microorganisms within pure cultures by analysing their infrared spectra; the sample set comprised prevalent pathogens commonly associated with human infections and sepsis [18]. They proposed an algorithm and method for creating a database of microbial FT-IR spectra, employing automated principal component analysis, and a comparison algorithm for identifying pathogenic microorganisms irrespective of culture conditions; testing on clinical *S. aureus* isolates reliably discriminated them from other infection-causing agents, including *E. faecalis*, *K. pneumoniae*, *E. coli*, *E. cloacae*, and *C. albicans*, in both pure cultures and mixed pairs. In recent years, there has been a growing interest in using FT-IR spectroscopy for the identification of bacterial pathogens relevant to sepsis [19]. Several studies have demonstrated the potential of FT-IR spectroscopy for the identification and differentiation of pathogenic bacteria [20,21,22]. However, most of these studies have focused on the identification of bacterial pathogens at the genus or species level, with limited information on sub-species identification and unrelated to sepsis (sepsis-irrelevant isolates).

In this study, we report the application of FT-IR spectroscopy combined with chemometrics for the classification and differentiation of clinically relevant invasive pathogens. Our focus diverges from previous studies that primarily examine laboratory isolates at the species and sub-species levels and aims to extend the applicability of FT-IR spectroscopy to clinical settings, specifically for the detection and characterisation of invasive pathogenic isolates in sepsis patients. Furthermore, we explore the capability of FT-IR spectroscopy in discriminating between two distinct groups of isolates from the same species, with a particular emphasis on identification and their specific antibiotic resistance characteristics. Our study not only enhances the utility of FT-IR spectroscopy as an effective tool for the detection and differentiation of sepsis-related bacteria but also highlights its potential for guiding targeted antibiotic therapies, addressing critical aspects in this field.

## 2. Materials and Methods

### 2.1. Chemicals, Bacterial Isolates, Growth Conditions, and Sample Preparation

All brain heart infusion (BHI) growth medium required for the study was acquired through a single purchase from Sigma Aldrich, thus ensuring the uniformity of the medium source throughout the experimental procedures. In this study, a total of 212 bacterial and fungal isolates were included, and their detailed information is provided in Table 1. The bacterial and fungal isolates were obtained from children diagnosed with invasive bacterial infection within the microbiology laboratory at Alder Hey NHS Foundation Trust, Liverpool, England. These isolates had been previously identified through clinical techniques including MALDI-TOF-MS. Initially, these isolates were received on Protect cryopreservative ceramic beads. Subsequently, at the Centre for Metabolomics Research (CMR) facility (UoL), the isolates were streaked onto BHI agar plates as three biological replicates and incubated at 37 °C for 18 h. Following the incubation period, the biomass from the surface of each plate was harvested using sterile inoculating loops and resuspended in 1 mL of sterile 0.9% sodium chloride (NaCl) solution. The optical density (OD_600_) of all samples was measured at 600 nm using a Jenway 6705 UV/Vis. spectrophotometer (Cambdridgeshire, UK). The samples were then centrifuged at 4 °C for 4 min at 5000× *g* using a benchtop Eppendorf microcentrifuge 5424R (Eppendorf Ltd., Cambridge, UK). The supernatant was discarded, and the biomass was washed by resuspending it in 1 mL of sterile physiological saline 0.9% NaCl solution, followed by another centrifugation step to eliminate any residues from the media. Finally, the bacterial concentration was adjusted to an OD_600_ of 20 (Bacterial cell cultures OD20 = 10^10^ CFU/mL). Quality control (QC) samples were prepared from a singular isolate originating from the CNS and were spotted onto all nine FT-IR plates to correct any plate-to-plate variation. The positions of QC samples on the FT-IR plate are illustrated in Figure S1.

❖ Details about the isolates, including any pertinent information regarding antibiotic resistance.
❖ The majority of CRE isolates originated from rectal swabs. *S. typhi* was obtained from a faecal sample, while a substantial proportion of both resistant and sensitive *Pseudomonas* isolates were derived from respiratory specimens. In addition, a significant portion of isolates identified as AHS, diphtheroid, *Bacillus*, and CNS were determined to be contaminants.

### 2.2. FT-IR Spectroscopy

A Bruker 96-well silicon sampling plate was cleaned by soaking it in sodium dodecyl sulfate (SDS) overnight, followed by multiple washing steps using 70% ethanol, and finally, it was rinsed with deionised water. Samples were prepared by spotting 20 µL aliquots (randomly) onto the clean plate followed by incubation to dryness at 42 °C for 45 min in a standard oven. A total number of 9 plates were used to analyse samples.

A Bruker Invenio infrared spectrometer (equipped with HTS-XT motorised microplate reader) was used to carry out the FT-IR spectroscopic analysis and collect the FT-IR spectral data in absorbance mode [23]. All spectra were obtained in the mid-infrared range between 4000 and 400 cm^−1^, with 64 scan co-adds at a 4 cm^−1^ resolution [24]. Prior to initiating the measurement process for each sample, the background spectra of the silicon substrate were collected. From each bacterial isolate, a total of 12 FT-IR spectra was collected, consisting of three biological and four analytical replicates. Three spots per sample and four readings from each spot were taken, resulting in a total of 12 spectra. The analysis time for the collection of these FT-IR spectra, with QCs, was 61 h (6.8 h per plate).

### 2.3. MALDI-TOF-MS

*Bacillus* species (five isolates) were identified using Matrix-Assisted Laser Desorption/Ionisation Time-of-Flight Mass Spectrometry (MALDI-TOF-MS; Bruker Daltonics GmbH & Co, Bremen, Germany). The direct transfer method was employed for sample preparation, wherein a single colony from a fresh overnight culture was touched with a toothpick, and a thin layer was smeared in duplicate onto the target plate as recommended by the manufacturer. Subsequently, 1 µL of 70% formic acid was applied, and after complete drying, the analyte was coated with 1 µL of Bruker HCCA matrix and then left to dry at room temperature before inserting it in the MALDI Biotyper^®^. Mass spectra were analysed with the MBT Compass software V4.1.100 (Bruker Daltonics).

### 2.4. Data Analysis

The FT-IR spectra were collected using the OPUS software V8.8, and they were subjected to pre-processing steps and multivariate analysis using MATLAB version R2020b (The Mathworks Inc., Natwick, MA, USA). The extended multiplicative signal correction (EMSC) algorithm was employed to scale the spectra [25]. Following this scaling procedure, the spectral region associated with CO_2_ vibrations (2400–2275 cm^−1^) was removed from the spectra and replaced with a linear trend. To minimise plate-to-plate variation, the spectra of all plates were then aligned toward a common target plate, which is plate 1. For each plate except plate 1, a calibration transfer model based on spectra subspace transformation [26] method was built between the current plate and plate 1 using QC spectra on the plates; this model was then applied to all the spectra on the current plate. The calibration transfer correction was performed using PLS Toolbox for MATLAB (Eigenvector Research, Inc., Manson, IA, USA).

PCA followed by PC-DFA was then conducted on the processed spectral data to extract and explore the clustering patterns and discriminate between different sample groups. PCA and PC-DFA are widely employed statistical methods in FT-IR spectroscopy for exploring data, recognising patterns, and performing classification. PCA reduces complex spectral data to uncorrelated variables called principal components (PCs), capturing the dataset’s most significant variation. These principal components were used as input for PC-DFA, aiming to minimise within-class variance while maximising between-class variance.

## 3. Results and Discussions

### 3.1. QC Correction and Data Alignment

**Figure 1:**
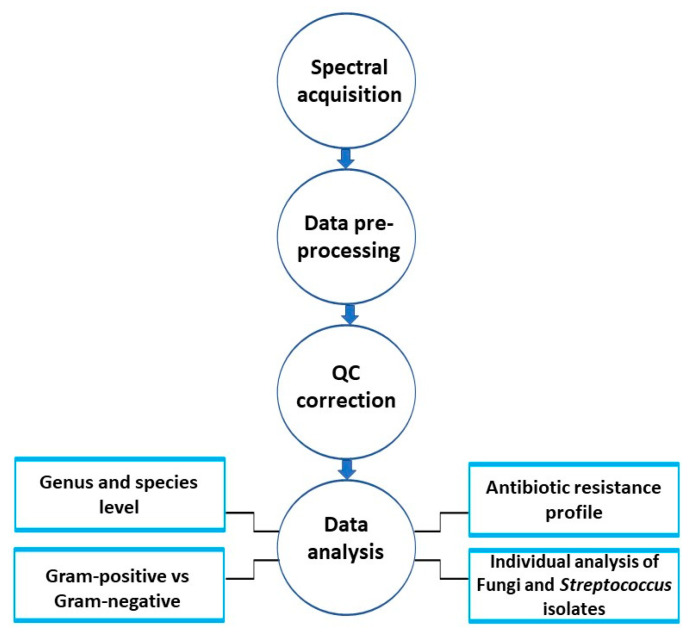
An overview of the FT-IR data analysis pipeline employed in this study.

**Figure 2:**
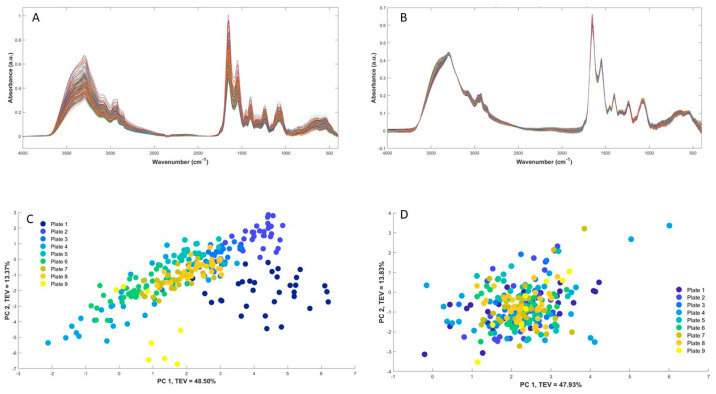
Quality control raw spectra (**A**), processed spectra (**B**), and PCA scores plot of QCs before calibration transfer (**C**) and after calibration transfer (**D**). TEV = total explained variance.

Following the acquisition of FT-IR spectral data, which took 61 h, a visual examination of the QC spectra was conducted as a preliminary step prior to conducting any analysis and aligning these infrared data collected on nine separate sample plates. This is crucial to check the quality of the data (adequate signal intensities), particularly with regard to the signal-to-noise ratio in the amide I region. An overview of the data acquisition and analysis pipeline employed in this study is provided in Figure 1. The FT-IR spectra obtained from the QC samples revealed a characteristic pattern consistent with intact bacterial spectra in Figure 2A,B. These fingerprints capture specific features that offer valuable insights into the composition and structure of biomolecules present within the microorganisms. Notably, the spectra prominently displayed absorbance bands primarily associated with the fatty acid and lipids in the 3000–2800 cm^−1^ range, proteins/peptides represented by amide I and amide II bands in the 1800–1500 cm^−1^ range, and the mix region involving phospholipids/DNA/RNA and phosphate-carrying compounds, providing information about proteins and fatty acids, in the 1500–1200 cm^−1^ range [27].

Additionally, the spectra exhibited absorption bands corresponding to polysaccharides present within the cell wall dominating the 1200–900 cm^−1^ range, along with the fingerprint region in the 900–700 cm^−1^ range, revealing unique and specific spectral patterns yet to be assigned to cellular components or functional groups [28]. Due to the large number of isolates investigated in this study, each with three biological replicates (and four technical replicates from each), the samples had to be analysed using nine different plates. Using multiple FT-IR plates for sample analysis introduces a higher likelihood of data variability between the plates. This can be attributed to various factors such as the environmental conditions of the FT-IR instrument (including temperature and humidity). These factors can significantly impact the quality and reproducibility of the collected spectral data. To remove such potential non-biological variations, following our previously published data processing pipeline [19], the QC samples were used to account for any plate-to-plate variations. QC samples carried critical information about between-plate variation and had been utilised to correct such variations by using the calibration transfer method. The result of QC correction is illustrated in Figure 2, where the PCA scores plot of the QC samples after alignment (Figure 2D) exhibit a significant improvement compared to using the spectral data before the alignment process (Figure 2C). This is apparent from the PCA scores plot of the initial spectral data, which clearly indicates a distinct separation of the QCs, despite them being essentially the same samples, based on their respective analysis batches (plates). However, in the aligned spectral data shown in Figure 2D, such variations are absent, and all QC samples appear to be uniformly mixed.

### 3.2. Classification and Discrimination of All Isolates

**Figure 3:**
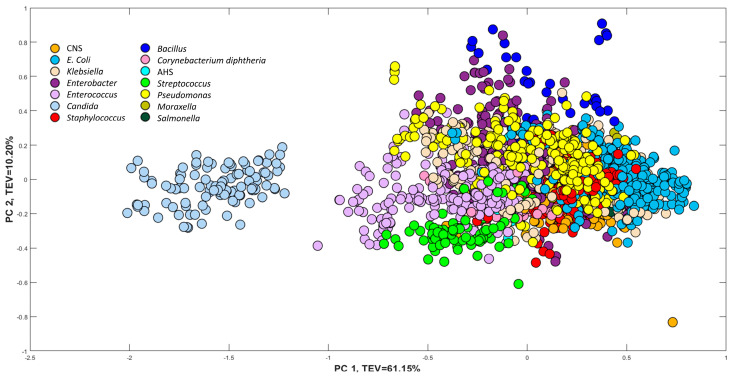
PCA scores plot of pre-processed spectral data of the organisms grown on BHI agar. The same species are plotted in a similar colour. TEV = total explained variance. Isolates belonging to the same species are represented using identical colours.

Following the QC correction, PCA was employed to explore the clustering patterns within the spectral data of all isolates. As depicted in Figure 3, the PCA scores plot showed that the most significant separation was between fungal and bacterial isolates, which is to be expected. The fungal species *Candida* is distinctly grouped on the far left side of the PC1 axis with a total explained variance (TEV) of 61.15% and separated from all other bacterial isolates.

To identify the biochemical features that significantly contributed to this separation, a PC1 loadings plot was generated (Figure S2), demonstrating dominant bands of 1657 cm^−1^ and 1547 cm^−1^ on the positive side of the PC1 axis, which are associated with proteins (specifically, amide I and II bands) [29]. In contrast, the most dominant band associated with fungal samples is positioned across the negative side of the PC1 loadings plot at 1045 cm^−1^, which is attributed to the vibrations of the carbohydrate backbone, suggesting that the carbohydrate composition plays a crucial role in distinguishing between fungi and bacteria. This can be attributed to their distinct cell wall compositions. Whilst fungal cells are characterised by a primary cell wall predominantly composed of glucans and chitin [30], a complex polysaccharide that imparts rigidity and structural support, bacterial cells possess a cell wall consisting of peptidoglycan, a unique polymer comprising sugars and amino acids. On the other hand, *Candida* spp. are known to produce carbohydrate structures (a slime layer) at the cell wall exterior with great efficiency, as reported in the literature [31].

Notably, the PCA scores plot also revealed that isolates sharing the same genera and species formed distinct clusters, indicating the discriminative potential of FT-IR spectroscopy in distinguishing microorganisms at the genus and species levels. This is particularly significant in clinical settings, where the precise identification and differentiation of microbial strains are crucial for accurate diagnosis, appropriate treatment selection, and epidemiological investigations.

**Figure 4:**
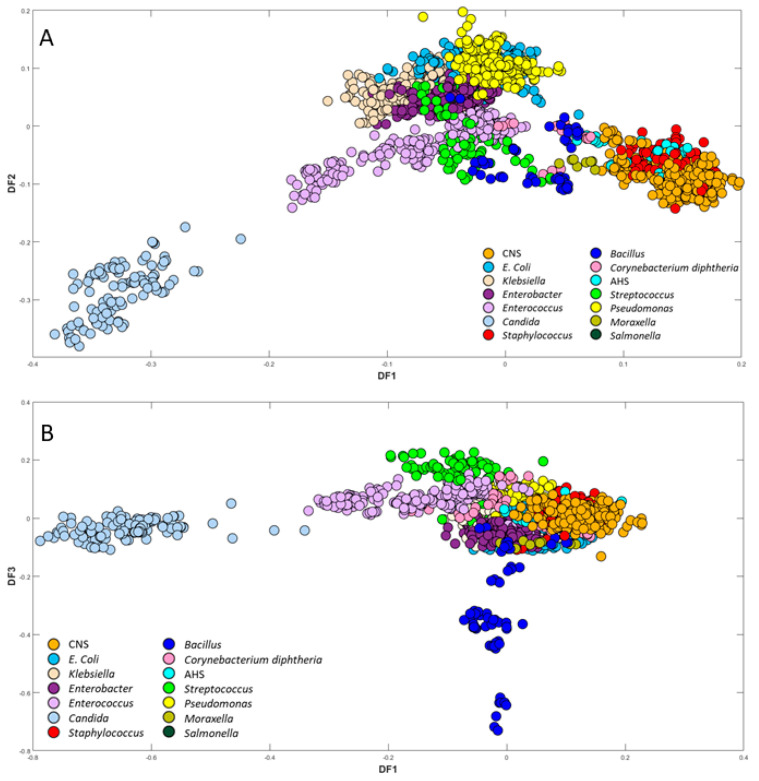
PC-DFA scores plot (20PCs, TEV = 98.72) of all isolates. The isolates of the same species are plotted in a similar colour (plot (**A**) is DF1 against DF2, and plot (**B**) is DF1 against DF3). PC-DFA is semi-supervised, and the class structure for DFA is based on biological replicates (3 biological replicates and 4 technical replicates) and is not the genera or species. Isolates belonging to the same species are represented using identical colours.

PC-DFA was then used as a semi-supervised method, with scores of the first 20 principal components (PC) as input data and the isolates as the class information, where 212 groups (i.e., isolates) were utilised as group information for classification; we call this semi-supervised in that the algorithm is only provided with information about the replicates and not the genus or species of a particular micro-organism. The PC-DFA scores plot (Figure 4) provided further separation between the bacterial samples, revealing 14 distinct clusters, demonstrating the effective grouping of the isolates from the same species, irrespective of their initial class assignments.

**Figure 5:**
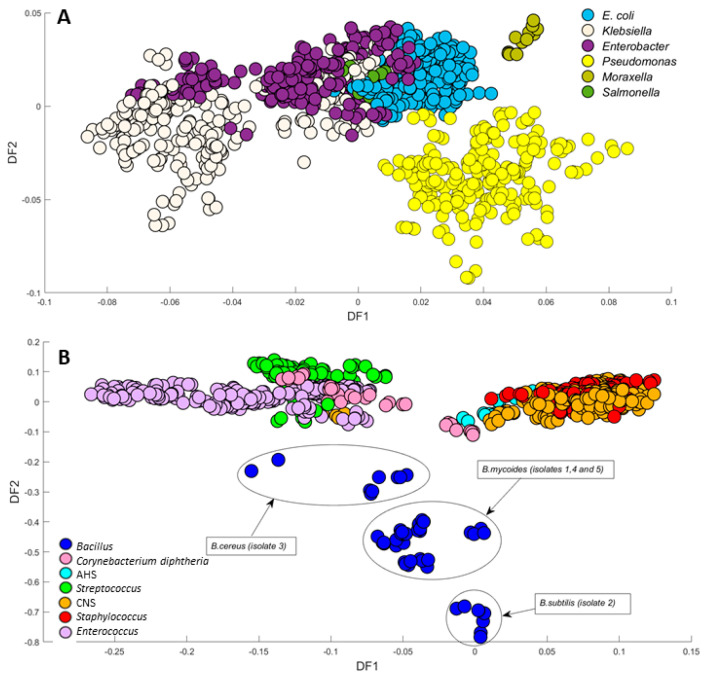
PC-DFA scores plot. (**A**) Gram-negative isolates (15PCs, TEV = 97.82%). (**B**) Gram-positive isolates (15PCs, TEV = 97.60%). The ellipses around the clusters on the PC-DFA scores plot indicate three different groups of Bacillus. PC-DFA is semi-supervised here, and the class structure for DFA is based on biological replicates (3 biological replicates and 4 technical replicates) and is not the genera or species. Isolates belonging to the same species are represented using identical colours.

The close grouping of certain clusters in the DFA scores plot can be attributed to the similarities observed in their spectral fingerprints. Notably, *Candida* spp. exhibited a clustering in the negative side of the DF1 axis, effectively separating it from the other isolates, which is consistent with the results obtained from the PCA scores plot. Furthermore, it is noteworthy that CNS and *Staphylococcus* clustered closely together towards the positive side of the DF1 axis, which is as expected since both belong to the same genus. In Figure 4B, *Bacillus* species were clustered separately from all other bacterial samples according to DF3 axis. Upon examining all FT-IR spectra, it was noted that the spectra of *Bacillus* species exhibited a prominent absorption band at 1737 cm^−1^, mainly attributed to ester vibrations (C=O stretching) [32]. As depicted in the pre-processed spectra (Figure S3A) and confirmed by the DF3 loadings plot (Figure S3B), the detected variation in the C=O stretching in saturated esters could be assigned to changes in the cellular lipid composition, including phospholipids and triglycerides [33,34]. This is perhaps not far from expected, as in *Bacillus* species, this C=O stretching vibration has been commonly linked to the presence of the lactam functional group with muramic acid, a characteristic structural component commonly found in endospores [35]. However, within the *Bacillus* species, three clustering patterns were observed. This discrepancy could be attributed to various factors, such as differences in the metabolic state of the *Bacillus* cells (growth phase), and potential sporulation. There was insufficient detailed information available concerning the distinct *Bacillus* isolates at the individual species level, with only a general reference to *Bacillus* species. Given the variations detected in *Bacillus* clustering patterns in the PC-DFA scores plot (Figure 4B and Figure 5B), and to address this concern comprehensively, we analysed all five *Bacillus* species using a Bruker MALDI Biotyper (MALDI Biotyper MSP Identification Standard Method 1.1) through the Extended Direct Transfer (eDT) Procedure to accurately identify them. The MADLI Biotyper findings indicated the existence of three different groups of *Bacillus* (annotated on this plot), specifically *B. mycoides*, *B. cereus*, and *B. subtilis*. This technique captures the unique proteomic fingerprint of a microorganism, matching the characteristic patterns with an extensive reference library. All samples achieved scores > 2, confirming the high confidence of identification. MALDI-TOF-MS presents a rapid, accurate, and cost-effective alternative to conventional methods. The ellipses around these clusters on the PC-DFA scores plot (Figure 5B) support the conclusions drawn from the FT-IR results and demonstrate the complete agreement between the MALDI-TOF-MS bacterial identification results and the FT-IR metabolic fingerprint analysis.

### 3.3. Differentiation between Isolates (Gram-Positive and Gram-Negative)

In order to further investigate the clustering patterns observed based on the FT-IR fingerprints, and to reduce the complexity of the data, all bacterial data were divided into two groups, Gram-positive and Gram-negative isolates.

Figure 5A illustrates a two-dimensional PC-DFA scores plot, where 15 principal components (PCs), accounting for 97.82% TEV, were utilised as input. While the plot demonstrated evident discrimination among all six distinct genera of Gram-negative bacteria, *Enterobacter* and *Klebsiella* displayed subclusters. This distinct clustering pattern arises from notable variances observed in the quantities and spatial distributions of essential cellular constituents, including nucleic acids, proteins, peptidoglycan, phospholipids, and lipopolysaccharides within these closely related bacterial species [36]. The significant impact of these major cellular components on the spectroscopic profiles facilitates a robust discrimination between the different bacterial groups.

Interestingly, the *Klebsiella* species clustering pattern appeared less tight than the other genera. This observation was anticipated and attributed to the presence of two distinct species, namely *K. oxytoca* and *K. pneumoniae*, within the *Klebsiella* genus. In order to investigate the distribution of clustering patterns observed among *Klebsiella* species, additional classifications were performed on these species: *K. oxytoca* and *K. pneumoniae*. The findings demonstrated discrimination between two *Klebsiella* species along the DF1 axes (Figure S4). This classification included six distinct groups that were utilised as input classes. These groups represented isolates of *K. oxytoca* and *K. pneumonia*, as well as both species that exhibited specific antibiotic resistance to Carbapenem-resistant Enterobacteriaceae (CRE) and Extended-spectrum β-lactamases (ESBL). Furthermore, it was observed that certain isolates of *K. pneumoniae* displayed a mucoid phenotype. In contrast, the remaining five groups each consist of a single species with a different number of isolates, potentially leading to their more closely tight clustering. Furthermore, within *E. coli*, *Enterobacter*, and *Klebsiella* groups, certain isolates were also found to possess specific antibiotic resistance, including ESBL and CRE. However, we were unable to differentiate these isolates based on their susceptibility to the respective antibiotics. Schaumann et al. achieved similar outcomes in their study when employing MALDI-TOF-MS protocol to differentiate between β-lactamase-negative strains of the species they examined (clinical isolates of *Enterobacteriaceae* and *P. aeruginosa*) and strains producing ESBLs and metallo-β-lactamases (MBLs). However, similar to our results, they concluded that the current reliability of this technique does not meet the standards required for routine diagnostic applications [37]. Similarly, in the case of *Pseudomonas*, there were isolates resistant to meropenem, but they could not be effectively discriminated from non-resistant *Pseudomonas* isolates. However, it is worth noting that all *Pseudomonas* isolates were separated from the rest of the samples according to DF2. Variations in specific spectral regions contributed to the differences between two Gram-negative bacteria, *P. aeruginosa* and *E. coli*. The highest spectral variations between *Pseudomonas* and other Gram-negative isolates were observed between 1800 cm^−1^ and 900 cm^−1^ as reported by Al-Qadiri et al. (2006). These spectral regions consist of the amide I band at 1650 cm^−1^ and the amide II band at 1550 cm^−1^. Additionally, they encompass the CH_3_ and CH_2_ asymmetric and symmetric deformations at 1455 cm^−1^ and 1398 cm^−1^, respectively, along with the stretching vibrations of polysaccharides ranging from 1200 cm^−1^ to 900 cm^−1^ [38]. Nevertheless, we employed the entire spectral region in the current investigation to distinguish between six Gram-negative isolates. The evident six distinguishable cluster groups along the DF1 axis in the PC-DFA indicate FT-IR spectroscopy’s efficacy with chemometrics in accurately discriminating Gram-negative isolates.

Similar to the Gram-negative isolates, a two-dimensional PC-DFA was carried out on the FT-IR spectral data collected from seven groups of Gram-positive bacterial isolates. The PC-DFA scores plot of these data revealed distinctive discrimination among the seven different groups (Figure 5B), where 15 PCs were employed, which accounted for 97.60% TEV. Contrary to the expected association with *Streptococcus* groups, the observed clustering of α-hemolytic *i* with coagulase-negative staphylococci (CNS) and *Staphylococcus* across the positive side of the DF1 axis prompts further investigation. The clustering might be influenced by the detected specific cell component, possibly the cell wall, suggesting similarities between AHS and other Gram-positive bacteria. As described earlier, the negative side of DF2 was dominated by *Bacillus* species, while all other microbial species were predominantly situated along the positive side of the DF2 axis. This notable separation is likely attributable to the influence originating from the ester carbonyl group (C=O) vibrational bond [32], as clearly evidenced in the plot of *Bacillus* spectra. Moreover, the DF2 loadings plot of the FT-IR data (Figure S3C) also further validated that the primary peak responsible for distinguishing *Bacillus* from all other Gram-positive species is associated with lipid vibrations at 1735 cm^−1^, which are attributed to the ester carbonyl group (C=O) vibrational bond. *Streptococcus* and *Enterococcus* species were distinctly clustered along the negative side of the DF1 axis, with *Streptococcus* exhibiting more compact clustering compared to *Enterococcus* (Figure 5B). Similar to the *Klebsiella* species within the Gram-negative section (Figure 5A), the *Enterococcus* species demonstrated heterogeneity and a more dispersed clustering of scores. The clustering patterns within the *Enterococcus* samples exhibit higher diversity when compared to *Streptococcus*. This diversity in clustering patterns was reasonably anticipated, considering that the presence of specific isolates was identified to possess pertinent antibiotic resistance characteristics within *Enterococcus* isolates, such as *Enterococcus faecalis*, *Enterococcus faecium*, and VRE species. This distinction was clearly evident through the implementation of PC-DFA analysis, where the scores plot effectively discriminates between the various *Enterococcus* species and VRE species.

### 3.4. Differentiation between Isolates According to Their Antibiotic Resistance Profile

#### 3.4.1. Differentiation between *S. aureus* Isolates

**Figure 6:**
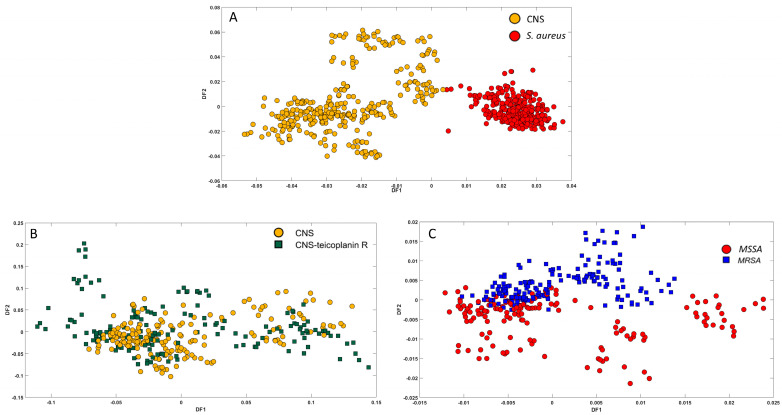
PC-DFA scores plot for (**A**) *S. aureus* and CNS isolates (15PCs, TEV = 98.89%), (**B**) CNS isolates and teicoplanin resistance (10Pca, TEV = 97.44%) and (**C**) MSSA and MRSA (30PCs, TEV = 99.34%). PC-DFA is semi-supervised here, and the class structure for DFA is based on biological replicates (3 biological replicates and 4 technical replicates) and is not the genera or species. Isolates belonging to the same species are represented using identical colours.

Due to the contrasting levels of virulence exhibited by *S. aureus* and CNS, the differentiation of these bacterial species in clinical samples has become a critical necessity [22]. Failure to accurately identify (misidentification) *S. aureus* as CNS may result in considerable expenses associated with determining the true cause of severe infections or resorting to unnecessary broad-spectrum empirical antibiotic treatments. Consequently, the precise identification of CNS isolates has become increasingly important for assessing their clinical significance and effectively managing CNS infection relapses. Figure 6A presents the PC-DFA scores plot, where 15 PCs (98.89% TEV) were utilised as input. The plot demonstrates a clear separation between CNS and *S. aureus* isolates along the DF1 axis, revealing two distinct clusters. Notably, the *S. aureus* isolates tightly cluster on the positive side of DF1, indicating a high degree of similarity among them. Conversely, the CNS isolates exhibit a more dispersed pattern of scores along the negative side of the DF1 plot, suggesting greater heterogeneity among these isolates. Remarkably, despite both *S. aureus* and CNS belonging to the same group, this FT-IR analysis demonstrated accurate discrimination between them. These findings were consistent with those obtained by Amiali et al., although their study focused on a narrow spectral region (2888–2868 cm^−1^), while ours utilised the full range of spectra for analysis [22].

The heterogeneity observed among CNS isolates may be attributed to the presence of teicoplanin resistance isolates among them. The effectiveness of FT-IR in differentiating between CNS and teicoplanin-resistant CNS was examined; however, the analysis did not yield successful discrimination between the two groups, as depicted in Figure 6B.

#### 3.4.2. MRSA vs. MSSA

To investigate the application of FT-IR spectroscopy in distinguishing between MRSA and MSSA within the *Staphylococcus* genus, 28 isolates of *S. aureus* were chosen for analysis using FT-IR spectroscopy. Although the *S. aureus* isolates depicted in Figure 6A clustered closely together, conducting PC-DFA analysis on the data from this species separately (Figure 6C) enabled the discrimination of the two groups of isolates based on the DF2 axis, utilising 30 PCs, accounting for 99.34% TEV. The separation observed between MRSA and MSSA can be attributed to the physicochemical properties of the biofilms produced by each isolate (the majority of MRSA strains were moderate biofilm producers) [39]. This variation has been correlated with the presence of the mecA gene [40]. Upon the loading plot for DF2 that was generated (Figure S5), it is evident that vibrations at 1076 cm^−1^ (P=O) and 1257 cm^−1^ are associated with vibrations along the sugar-phosphate chain (linked to biofilm production) and the conformation of the nucleic acid backbone. Although the separation between the two groups was not ideal, the implementation of FT-IR with chemometrics in the investigation of 28 bacterial clinical isolates and the observed separation between MRSA and MSSA, even without antibiotic challenge, demonstrated highly promising and encouraging results. This suggests the considerable potential of this technology in clinical microbiology, highlighting a valuable direction for future research. The findings are in agreement with a prior study conducted by Suntsova et al. that aimed to predict MRSA and MSSA phenotypes in 20 *S. aureus* isolates based on their FT-IR spectra. Nonetheless, using a larger sample size indicates that our study’s findings are more reliable and statistically robust due to the increased number of samples [18].

#### 3.4.3. *Enterococcus* Species and VRE

**Figure 7:**
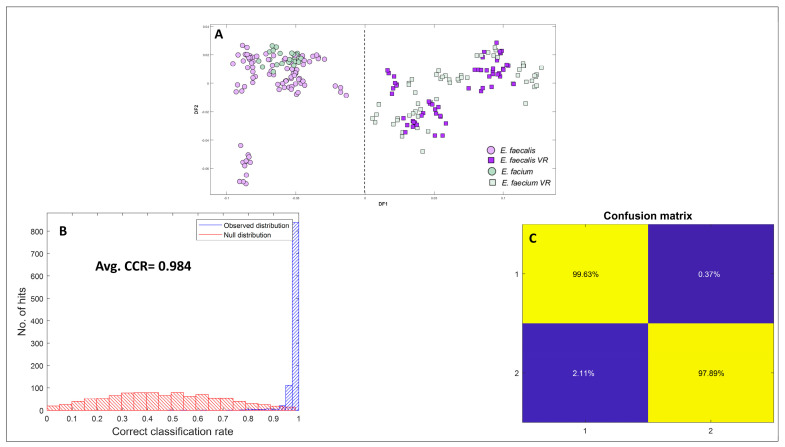
PC-DFA scores plot and PLS-DA results for a model classifying bacteria with subjects divided into two groups: (class 1 = enterococcus species, class 2 = VRE species). (**A**) Enterococcus and VRE species (15PCs, TEV = 91.68%), PC-DFA is semi-supervised here and the class structure for DFA is based on biological replicates (3 biological replicates and 4 technical replicates) and is not the genera or species. Isolates belonging to the same species are represented using identical colours, (**B**) PLS-DA bootstrap model’s null distribution (red) and observed distribution (blue) are presented alongside the overall correct classification rate (CCR). (**C**) Displays the confusion matrix showing observed correct classifications for each group individually based on validated PLS-DA.

Another encouraging finding in this study is the ability to effectively differentiate between *Enterococcus* species and VRE with a notably high level of accuracy. This successful discrimination was clearly evident in the generated PC-DFA scores plot, as depicted in Figure 7A, where 15 PCs, accounting for 91.68% TEV, were utilised as inputs. To investigate the underlying factors contributing to the differentiation between *Enterococcus* and VRE achieved through PC-DFA, we calculated the average spectra of *Enterococcus* and VRE, as illustrated in Figure S5A. The plot clearly indicated that differences between these two groups may arise from variations in lipids and phosphate contents. The peaks at 3300 cm^−1^, 2922 cm^−1^, and 1078 cm^−1^ are the most prominent spectral changes observed in VRE isolates. They correspond to the fatty acid and lipid region, as well as the P=O stretching vibration of phosphate potentially associated with nucleic acids and polysaccharides [41]. The differentiation between the two groups was likely attributed to the presence of cell wall teichoic acid (WTA), peptidoglycan, and lipoteichoic acid. Notably, the occurrence of lipoteichoic acid could be linked to the presence of thicker cell walls, a characteristic typically observed in the envelope of Gram-positive bacteria [42]. To strengthen our interpretation, a standard purified lipoteichoic acid (LTA) was analysed using FT-IR spectroscopy within the wavelength range of 4000–400 cm^−1^, and its spectrum was collected to verify if the spectral bands align with the conclusions drawn from the averaged spectra (Figure S6A). The spectral data from LTA revealed the presence of an O–H stretching of hydroxyl groups vibration around 3300 cm^−1^ (Figure S6B). The peak at 2924 cm^−1^ was attributed to the C–H stretching (asymmetric) in fatty acid chains. Additionally, weak bands observed around 1654 cm^−1^ were associated with the C=O bonds. Moreover, the FT-IR spectrum exhibited an absorption band at 1220 cm^−1^, indicating the P=O stretching (asymmetric) of PO_2_ phosphodiesters. Finally, the peak observed near 1050 cm^−1^ indicated the C–C and C–OH ring vibrations of carbohydrates [43]. The comparative analysis of the structural characteristics of LTA with the loadings plot generated between VRE and *Enterococcus* species revealed a substantial overlap. The presence of key functional groups and biochemical components observed in the LTA spectrum significantly coincided with the positive axis of DF1 loadings plot. This outcome provided substantial support for the hypothesis that discrimination between VRE and *Enterococcus* species can be potentially attributed to the presence of LTA in the cell wall of VRE isolates.

To evaluate the accuracy of classification models (derived from PC-DFA) in distinguishing between *Enterococcus* spp. and VRE, a partial least squares-discriminant analysis was used as a supervised learning method for discrimination. In this process, training data pairs (FT-IR spectra and known identities: *Enterococcus* spp. vs. VRE encoded as (e.g.,) ‘1’ and ‘2’) are used to calibrate PLS-DA models. Once calibrated, these can be challenged with FT-IR spectra and a prediction can be made. In order to validate this process, 1000 bootstrapping validations were utilised, along with permutation testing (where the Y-variable is randomised) as described by Gromski et al. [44]. Figure 7B shows the predictions from the 1000 test sets with the true test sets highlighted in blue, with the null distributions from permutation testing in red. The method also generated a confusion matrix (Figure 7C) that demonstrates a notable high accuracy in correctly classifying observations for each group separately. The confusion matrix is an essential tool in machine learning for assessing classification models by providing detailed insights into predictions such as true positive, true negative, false positive, and false negative instances. True positives and true negatives represent accurate predictions, while false positives and false negatives indicate prediction errors. These elements are used to calculate performance metrics like accuracy which evaluates overall correctness based on the relationship between correct predictions and total outcomes. Figure 7B displays the observed and null distributions obtained from bootstrap validation for the PLS-DA models, with an average correct classification rate of 0.984 and a *p*-value < 0.001. Each histogram is accompanied by the corresponding averaged confusion matrix for these models Figure 7C. This outcome validates the accuracy of the classification model in utilising FT-IR as a diagnostic tool for discrimination between *Enterococcus* and VRE, as it achieves over 98% accuracy in prediction.

### 3.5. Discrimination and Classifying Streptococcus and Candida Species

**Figure 8:**
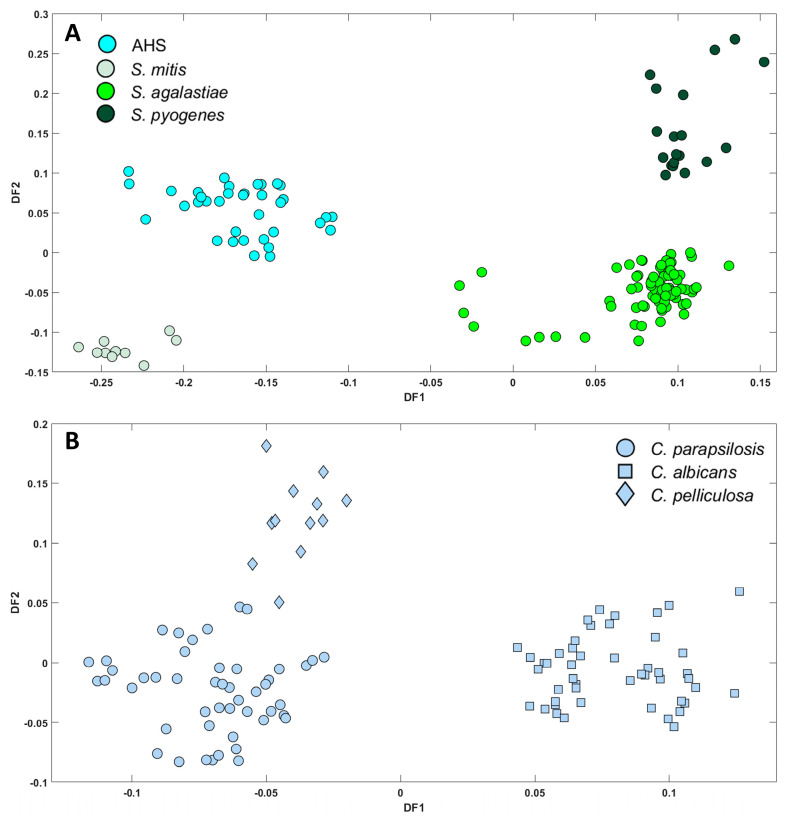
PC-DFA scores plot for (**A**) four different species of Streptococcus isolates (10PCs, TEV = 97.26%) and (**B**) three different Candida species (10PCs, TEV = 97.08%). PC-DFA is Semi-supervised here and the class structure for DFA is based on biological replicates (3 biological replicates and 4 technical replicates) and is not the genera or species. Isolates belonging to the same species are represented using identical colours.

To assess the discriminatory potential of FT-IR in distinguishing between different species within the same genera, *Streptococcus* and *Candida* species were subjected to PC-DFA separately. Figure 8A illustrates the PC-DFA scores plot for *Streptococcus* isolates (15 PCs as inputs accounting for 89.21% TEV). The plot revealed four distinct groups, AHS, *S. agalactiae*, *S. mitis*, and *S. pyogenes*, represented by different colours. Despite the class structure, the semi-supervised method demonstrated its capability to identify successfully seven species of *S. agalactiae* and group them together, with three biological replicates consistently placed in the same class rather than genera or species. Similar results were obtained for *S. pyogenes* and AHS, with different class structures showing consistent classification. Nonetheless, when considering *S. mitis*, which was the sole species identified within the AHS category and was assigned to a separate class, although positioned in close proximity to the AHS cluster, it displayed some differences. To identify the biochemical differences between AHS and *S. mitis*, a DF2 loading plot was generated to show vibrational regions with spectral changes Figure S7B. Increased biochemical changes were observed in the fatty acid chains, lipids, nucleic acids, and polysaccharides regions of *S. mitis* Figure S7A (negative part of DF2 axis), possibly contributing to the differentiation between the two groups. In the study by Van der Mei et al., distinct species clusters were observed during the analysis of *Streptococcus* species. However, it is noteworthy that among the 12 isolates of *S. mitis* examined, 3 isolates exhibited a distinct positioning outside the conventional species cluster. Instead, they formed a combined cluster along with *S. sanguinis* and *S. gordonii* [45].

A similar approach was employed to distinguish between the 10 isolates belonging to three different *Candida* species (*C. albicans*, *C. glabrata*, and *C. parapsilosis*). The spectral data from these samples were subjected to PC-DFA, where 10 PCs, accounting for 97.08% TEV, were utilised as inputs. In contrast to *Streptococcus* species, which displayed noticeable differences in their infrared spectra within their respective species (Figure S7A), the mean infrared spectra of the three *Candida* species indicated remarkable similarity (Figure S8A). Despite the similarities observed in the infrared spectra of the three *Candida* species, our PC-DFA scores plot (Figure 8B) revealed the existence of three distinct groups, thus confirming the applicability of FT-IR in differentiating *Candida* at the species level. Upon generating the DF1 loadings plot Figure S8B, significant differences were observed in the regions corresponding to carbohydrates, phospholipid/RNA/DNA, and protein (amide II) from 1225 to 975 cm^−1^ and 1596 cm^−1^. These dissimilarities are commonly associated with DNA and RNA C–O stretching as well as carbonyl (C=O). This finding is consistent with the results obtained by Timmins et al. [46] and Silva et al. [47]. Meanwhile, in the later study, FT-IR-ATR coupled with chemometrics enabled the successful discrimination of five prevalent *Candida* species, along with an additional 12 closely related and less common *Candida* species [47].

## 4. Study Limitation

The limitation of our study lies in the inability of FT-IR to differentiate certain species, such as *E. coli*, *Klebsiella*, *Enterobacter*, and *Pseudomonas*, based on their resistance profile against ESBL, CRE, and meropenem-resistant *Pseudomonas*. Nonetheless, the research lays a robust groundwork for the extended application of FT-IR and chemometrics in clinical and microbiological applications.

In the future, we shall also assess challenging these bacteria with antibiotics and assessing the effects on the bacterial phenotype to see if this enhances antibiogram testing using infrared spectroscopy, as illustrated very recently by us for the AMR profiling of *E. coli* at the single-cell level [17]. This approach can also elucidate the modes of resistance, as shown using Raman spectroscopy on *P. aeruginosa* challenged with the aminoglycoside antibiotic amikacin [48].

## 5. Conclusions

In conclusion, this study demonstrates the successful application of infrared spectroscopy, combined with chemometrics, as a powerful tool for classifying and identifying microbial species in sepsis patients. We achieved high reproducibility in clustering patterns between genera by analysing 212 isolates, consisting of 202 bacteria and 10 fungi. The incorporation of quality control samples and the use of a semi-supervised method of PC-DFA further enhanced the reproducibility of the data.

It is important to emphasise that our study successfully demonstrated the capability of FT-IR to differentiate between isolates of the same species, wherein specific isolates were identified to possess pertinent antibiotic resistance characteristics. However, it is worth noting that FT-IR was unable to distinguish between certain species, such as *E. coli*, *Klebsiella*, *Enterobacter*, and *Pseudomonas*, with respect to their resistance against ESBL (Extended-spectrum β-lactamases), CRE (Carbapenem-resistant Enterobacteriaceae), and meropenem-resistant *Pseudomonas*.

The application of PCA revealed notable clustering patterns between the different genera, and the subsequent implementation of PC-DFA resulted in increased reproducibility and clear discrimination among the groups. Furthermore, CNS and *S. aureus* isolates were accurately discriminated, and promising results were achieved in distinguishing *S. aureus* from MRSA.

Of particular interest was the remarkable discrimination achieved between *Enterococcus* and VRE isolates with an accuracy of over 98% when the validation model was implemented. Moreover, the different *Streptococcus* and *Candida* samples were discriminated down to species level. These accomplishments further underscore the potential of FT-IR as a valuable technique for investigating microorganisms, highlighting its sensitivity and specificity.

Overall, these techniques offer significant potential in diagnosing and managing invasive infections through advanced microbial identification, thereby leading to improved patient outcomes.

## Figures and Tables

**Table 1:** Microorganisms analysed in this study. Each species is represented by a different colour.

Organisms Name	No of Isolates	Name of Isolates	Colour
AHS	3	Alpha-hemolytic streptococci	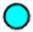
*Bacillus*	5	*Bacillus* species	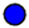
*Candida*	10	*C. parapsilosis, C. albicans and C. pelliculosa*	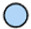
CNS	28	Coagulase-negative staphylococci and teicoplanin resistant CNS	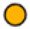
*Corynebacterium diphtheriae*	4	Diphtheria	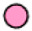
*E. coli*	27	*E. coli*, ESBL: Extended-spectrum β-lactamases, CRE: Carbapenem-resistant Enterobacteriaceae	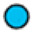
*Enterobacter*	26	*E. cloacae*, CRE and ESBL	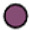
*Enterococcus*	20	*E. faecalis*, *E. faecium*, other *Enterococcus* species and VRE (Vancomycin-resistant enterococci)	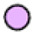
*Klebsiella*	26	*K. oxytoca* and *K. pneumoniae*, CRE and ESBL	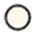
*Moraxella*	1	*Moraxella* species	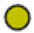
*Pseudomonas*	22	*P. aeruginosa* and meropenem resistant *P. aeruginosa*	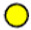
*Salmonella*	1	*S. typhi*	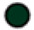
*Staphylococcus*	28	*S. aureus* and MRSA (Methicillin-resistant *Staphylococcus aureus*)	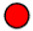
*Streptococcus*	11	*S. mitis*, *S. pyogenes* and *S. agalactiae*	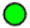
Total	212		

## Data Availability

All data generated in this study will be made available upon request.

## Supplementary Materials

The following supporting information can be downloaded at: https://www.mdpi.com/article/10.3390/microorganisms12071415/s1, Figure S1: Spotting Pattern of Quality Control (QC) samples on The FT-IR plate. B is the blank and used as the reference background spectrum for each plate; Figure S2: PC1 loadings plot of FT-IR spectral data of all isolates. This is linked to Figure 3 in the main text; Figure S3: (A) Averaged pre-processed spectra for Bacillus species and (B) DF3 loadings plot of FT-IR spectral data of all isolates (C) DF2 loadings plot of FT-IR spectral data of Gram positive isolates. This is linked to Figure 4B and Figure 5B in the main text; Figure S4: PC-DFA score plot for K. oxytoca and K. pneumoniae isolates (15PCs, TEV=98.24%); Figure S5: DF2 loadings plot of FT-IR spectral data of MRSA and MSSA isolates. This is linked to Figure 6C in the main text; Figure S6: The FT-IR spectra of Lipoteichoic acid (A) and (B) Averaged FT-IR spectral data of Enterococcus species and VRE. This is linked to Figure 7A in the main text; Figure S7: (A) Averaged spectra for Streptococcus species and (B) DF2 loadings plot of FT-IR spectral data of Streptococcus species. This is linked to Figure 8A in the main text; Figure S8: (A) Averaged spectra for Candida species and (B) DF1 loadings plot of FT-IR spectral data of Candida species. This is linked to Figure 8B in the main text.
