# Supplementary material for: Rapid Classification and Differentiation of Sepsis-Related Pathogens Using FT-IR Spectroscopy
Source: Microorganisms. 2024 Jul 12;12(7):1415. doi: 10.3390/microorganisms12071415 (PMC11279078; doi:10.3390/microorganisms12071415)
Supplement: Supplementary file 1 [file microorganisms-12-01415-s001.zip › microorganisms-3060862-supplementary.pdf]

Rapid Classification and Differentiation of Sepsis-related Pathogens Using FT-IR Spectroscopy

Shwan Ahmed [1,4], Jawaher Albahri [1,5], Sahand Shams [1], Silvana Sosa-Portugal [6], Cassio Lima [1], Yun Xu [1], Rachel McGalliard [2], Trevor Jones [2], Christopher M Parry [3], Dorina Timofte [6], Enitan D Carrol [2], Howbeer Muhamadali [1*], Royston Goodacre [1*]

[1] Centre for Metabolomics Research, Department of Biochemistry, Cell and Systems Biology, Institute of Systems, Molecular and Integrative Biology, University of Liverpool, Liverpool, L69 7ZB, United Kingdom; shwan.ahmed@liverpool.ac.uk; j.albahri@ liverpool.ac.uk; sahand.shams@ liverpool.ac.uk; cassio.lima@ liverpool.ac.uk; yun.xu@ liverpool.ac.uk
[2] Department of Clinical Infection, Microbiology and Immunology, Institute of Infection, Veterinary and Ecological Sciences, University of Liverpool, Liverpool, L69 7BE, United Kingdom; rmcg@ liverpool.ac.uk; trjones@ liverpool.ac.uk; edcarrol@ liverpool.ac.uk
[3] Department of Clinical Sciences, Liverpool School of Tropical Medicine, Liverpool, UK; christopher.parry@lstmed.ac.uk
[4] Department of Environment and Quality Control, Kurdistan Institution for Strategic Studies and Scientific Research, Sulaymaniyah, Kurdistan Region, Iraq
[5] Department of Pharmaceutical Chemistry, College of Pharmacy, King Khalid University, Abha 62529, Saudi Arabia
[6] Department of Veterinary Anatomy, Physiology and Pathology, Institute of Infection, Veterinary and Ecological Sciences, University of Liverpool, Neston, United Kingdom; s.sos-portugal@ liverpool.ac.uk; tdorina@ liverpool.ac.uk
***** Correspondence: roy.goodacre@liverpool.ac.uk and howbeer.muhamad-ali@liverpool.ac.uk

Supplementary

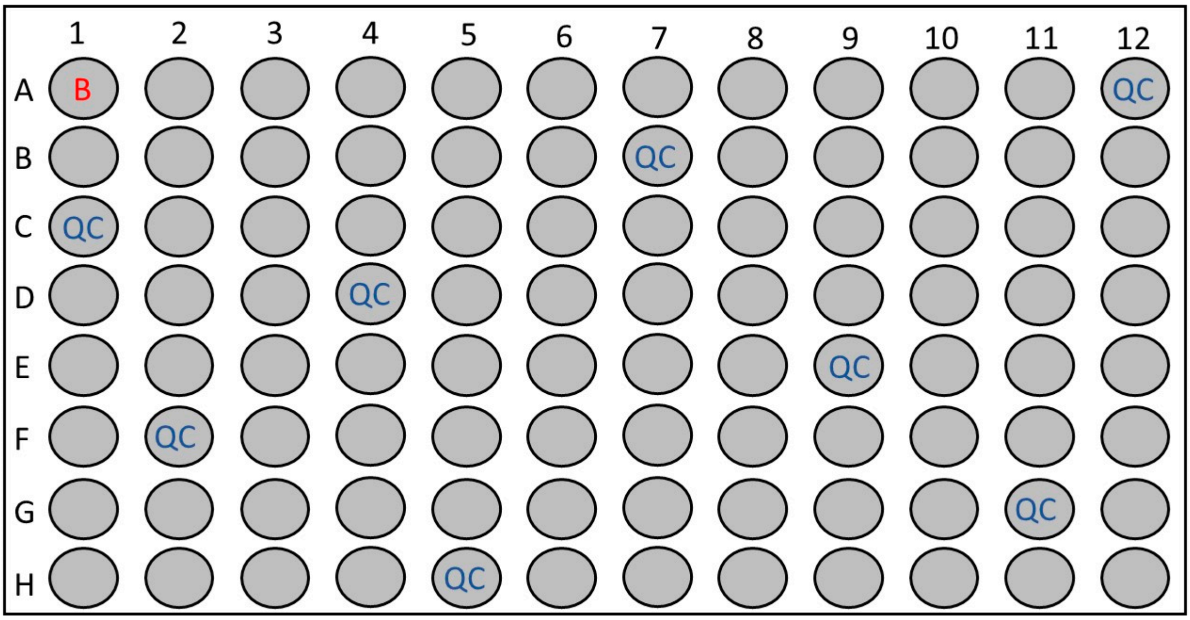

Figure S1 Spotting Pattern of Quality Control (QC) samples on The FT-IR plate. B is the blank and used as the reference background spectrum for each plate.

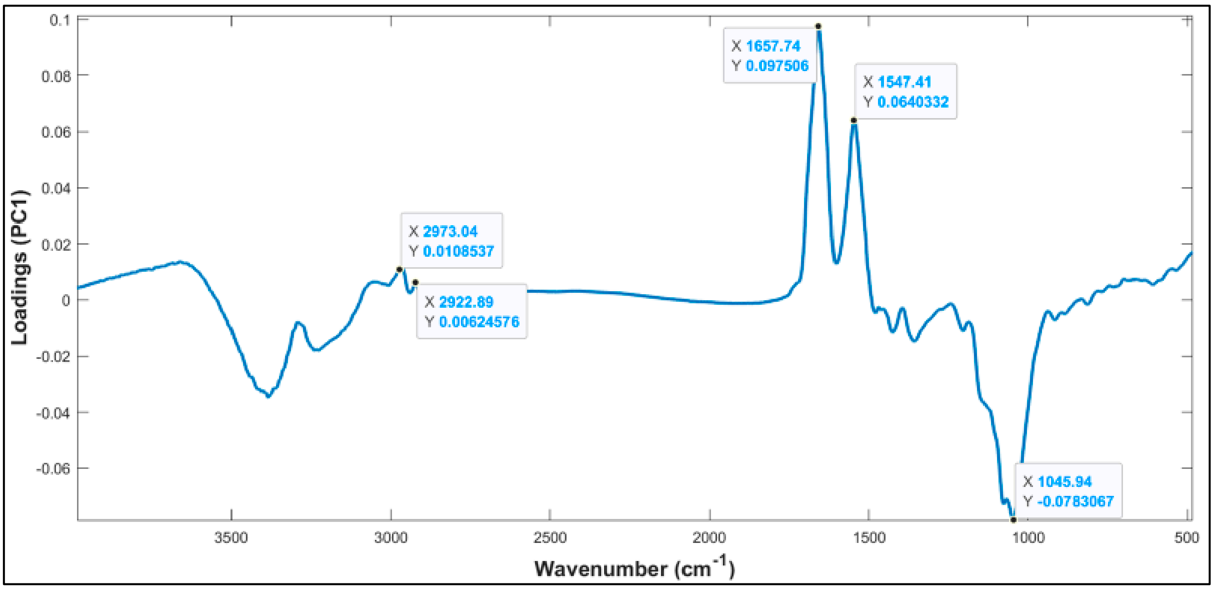

Figure S2 PC1 loadings plot of FT-IR spectral data of all isolates. This is linked to Figure 3 in the main text

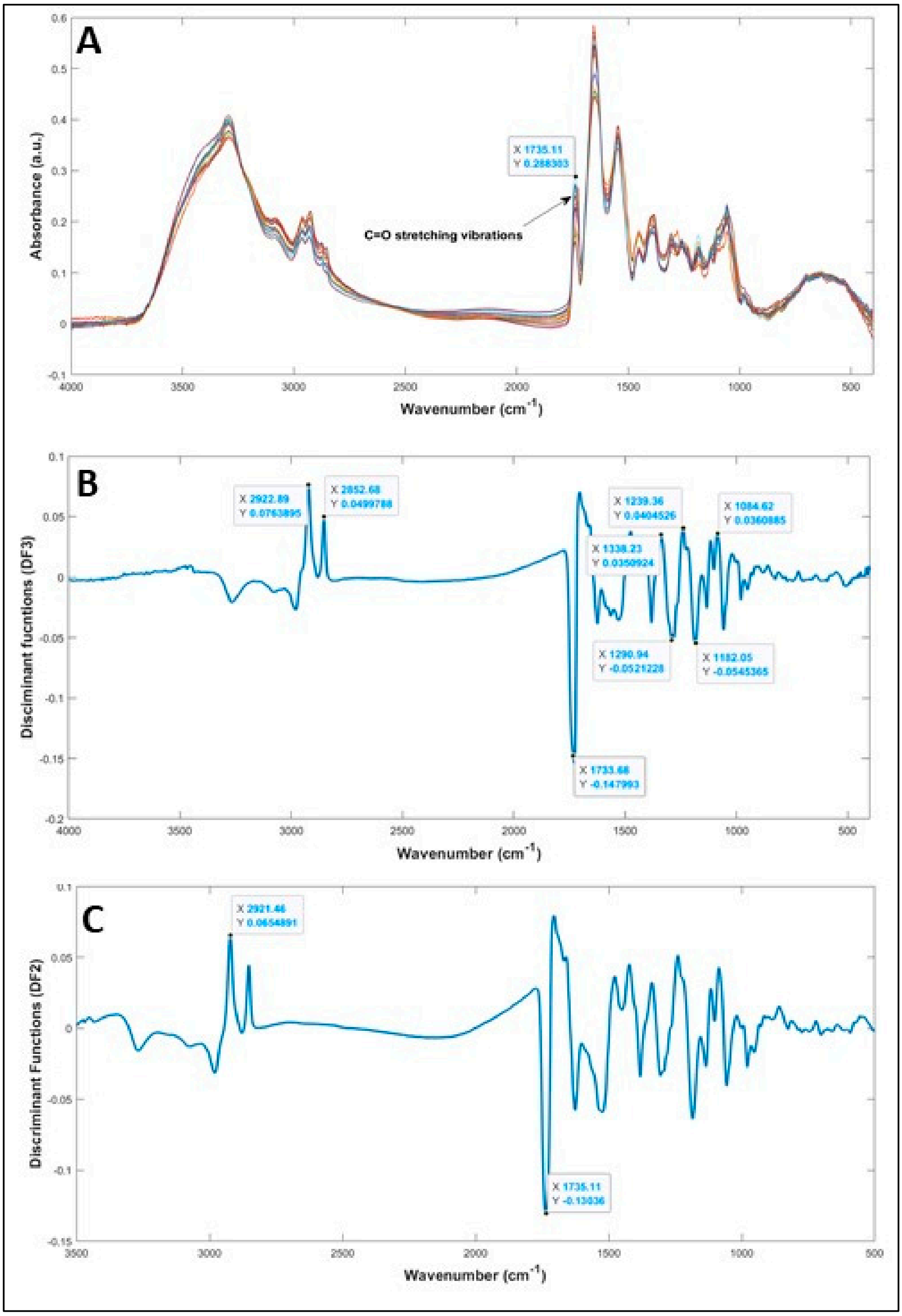

Figure S3 A) Averaged pre-processed spectra for Bacillus species and B) DF3 loadings plot of FT-IR spectral data of all isolates C) DF2 loadings plot of FT-IR spectral data of Gram positive isolates. This is linked to figure 4B & 5B in the main text.

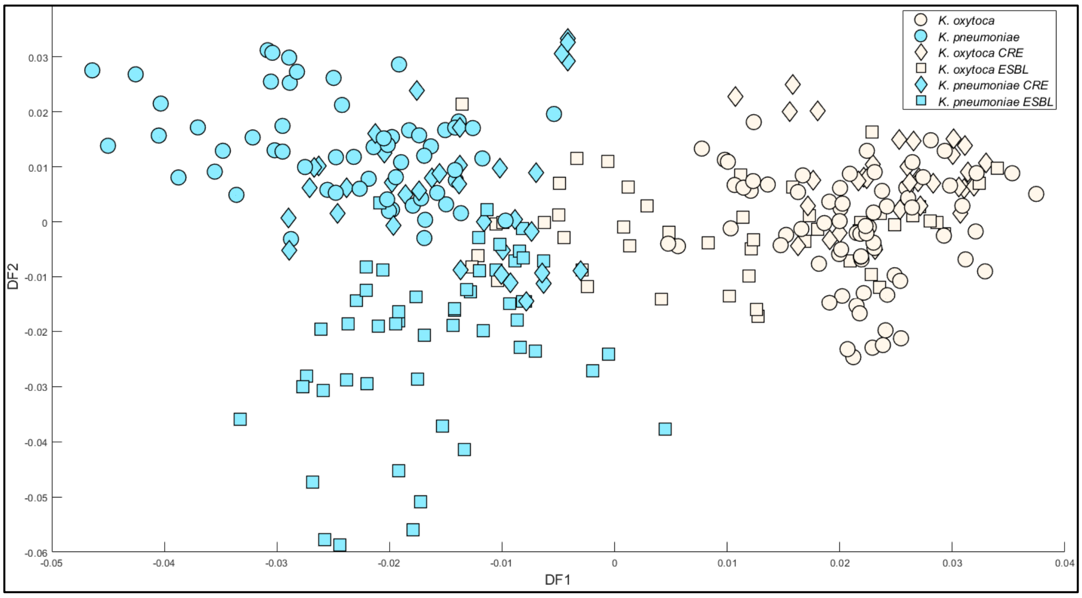

Figure S4 PC-DFA score plot for K. oxytoca and K. pneumoniae isolates (15PCs, TEV=98.24%)

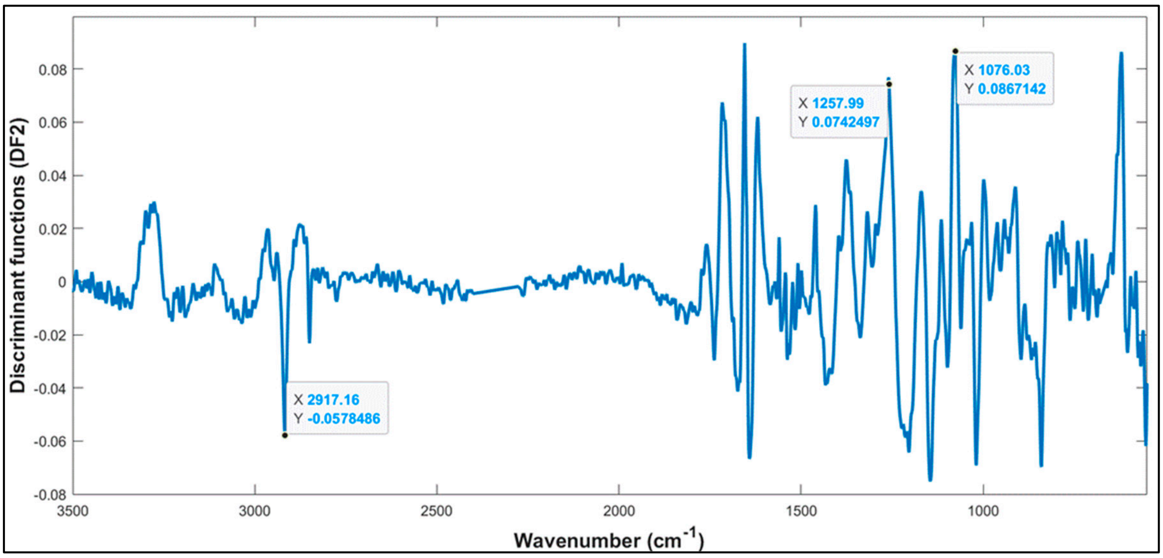

Figure S5 DF2 loadings plot of FT-IR spectral data of MRSA and MSSA isolates. This is linked to figure 6C in the main text.

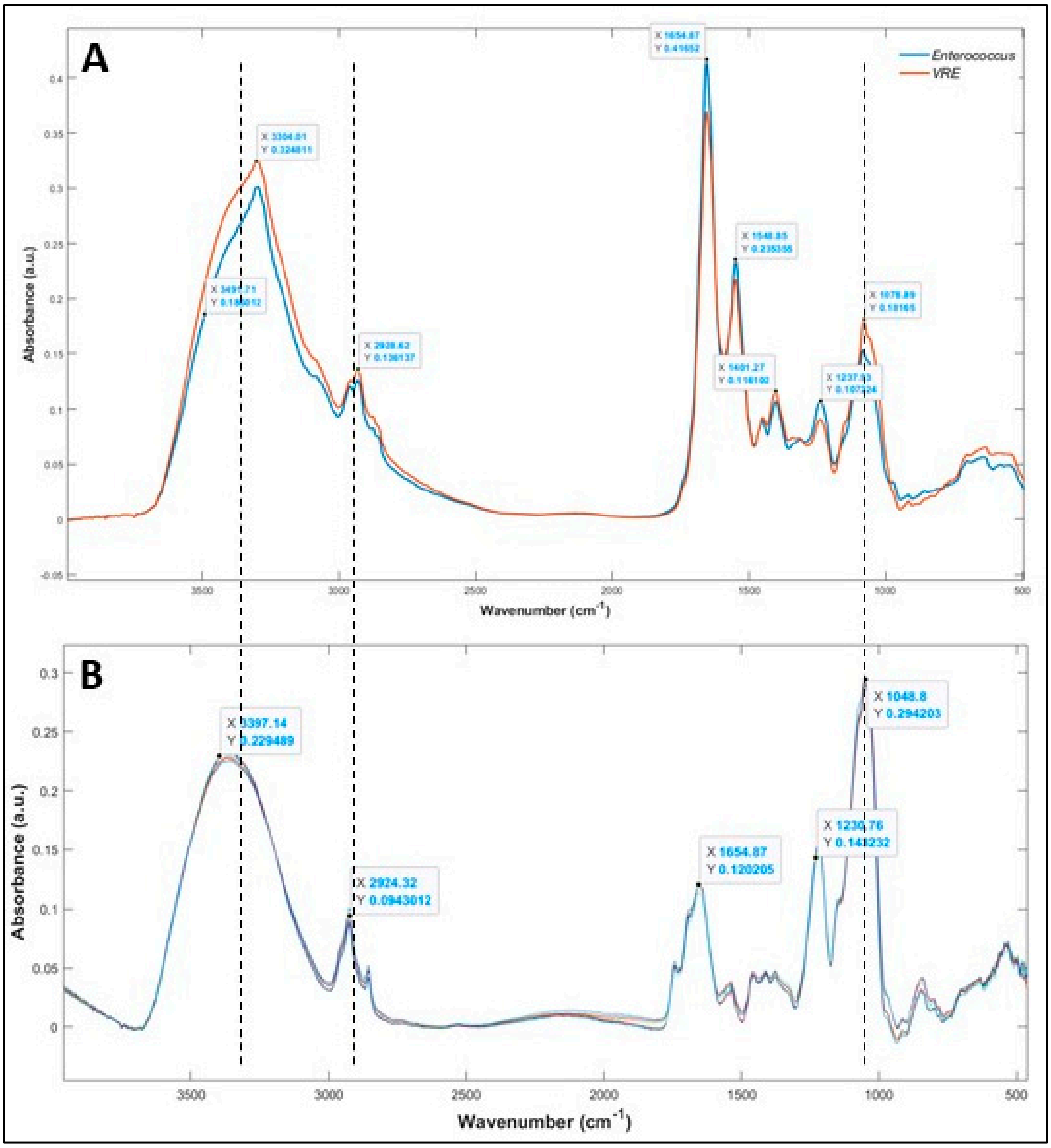

Figure S6 The FT-IR spectra of Lipoteichoic acid A) and B) Averaged FT-IR spectral data of Enterococcus species and VRE. This is linked to figure 7A in the main text.

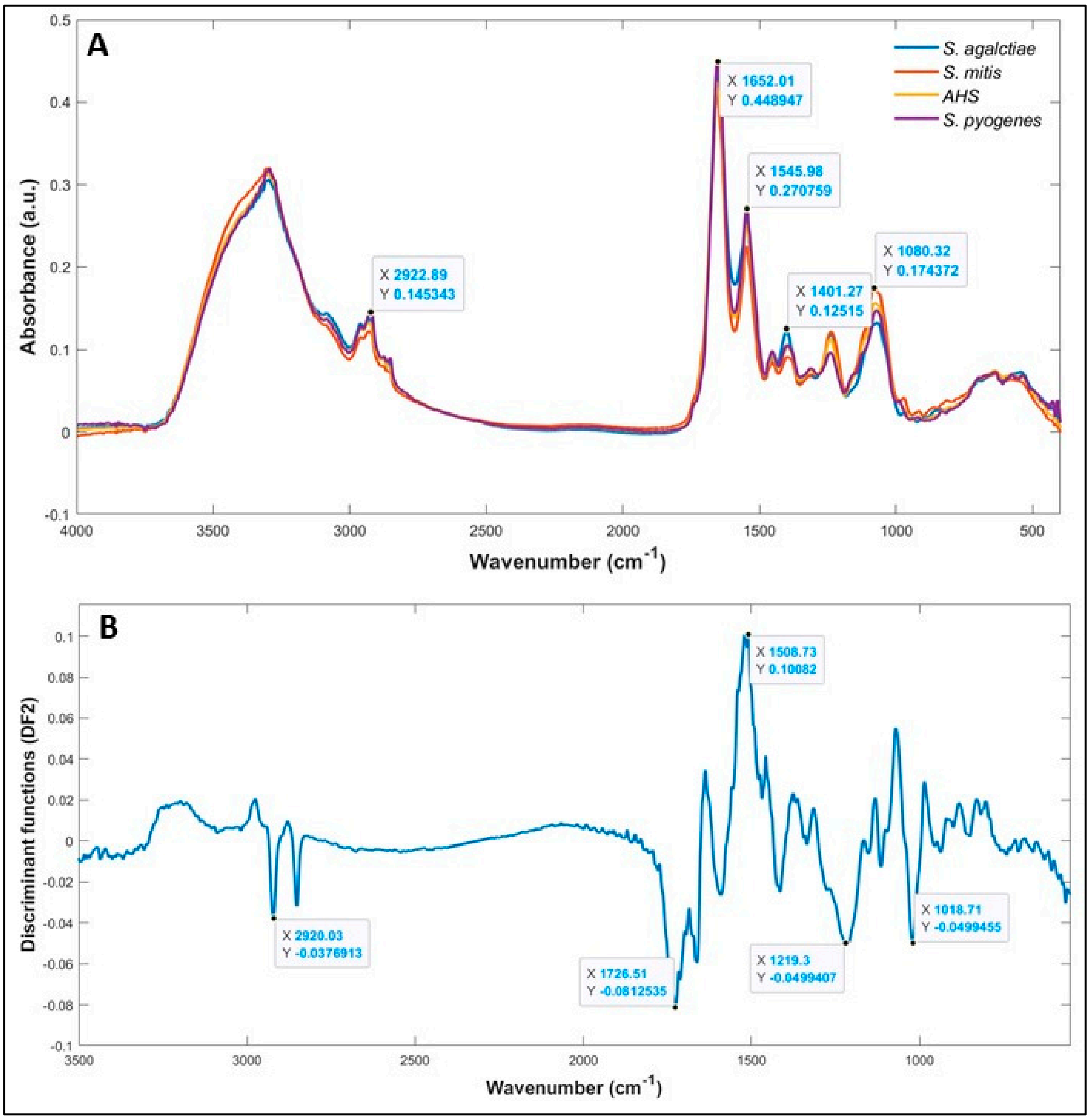

Figure S7 A) Averaged spectra for Streptococcus species and B) DF2 loadings plot of FT-IR spectral data of Streptococcus species. This is linked to figure 8A in the main text.

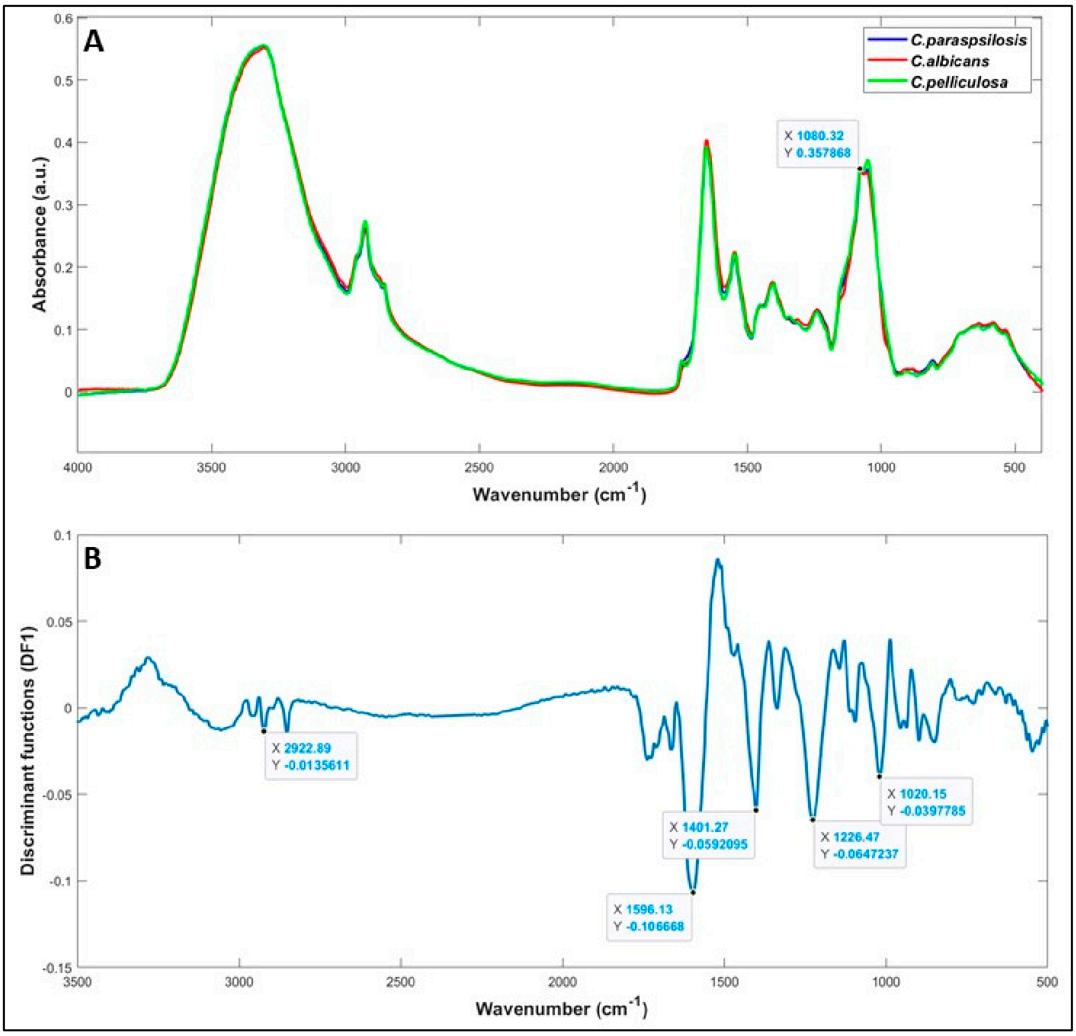

Figure S8 A) Averaged spectra for Candida species and B) DF1 loadings plot of FT-IR spectral data of Candida species. This is linked to figure 8B in the main text.
